# *HvGSK1.1* Controls Salt Tolerance and Yield through the Brassinosteroid Signaling Pathway in Barley

**DOI:** 10.3390/ijms25020998

**Published:** 2024-01-13

**Authors:** Yuliya Kloc, Marta Dmochowska-Boguta, Paulina Żebrowska-Różańska, Łukasz Łaczmański, Anna Nadolska-Orczyk, Wacław Orczyk

**Affiliations:** 1 Plant Breeding and Acclimatization Institute—National Research Institute, Radzikow, 05-870 Blonie, Poland; m.dmochowska-boguta@ihar.edu.pl (M.D.-B.); a.orczyk@ihar.edu.pl (A.N.-O.); w.orczyk@ihar.edu.pl (W.O.); 2 Laboratory of Genomics and Bioinformatics, Hirszfeld Institute of Immunology and Experimental Therapy, Polish Academy of Sciences, 53-114 Wrocław, Poland; paulina.zebrowska-rozanska@hirszfeld.pl (P.Ż.-R.); lukasz.laczmanski@hirszfeld.pl (Ł.Ł.)

**Keywords:** bikinin, 24-epibrassinolide (EBL), biomass, brassinosteroids, BR signaling pathway, CRISPR/Cas9, kernel weight, salt stress

## Abstract

Brassinosteroids (BRs) are a class of plant steroid hormones that are essential for plant growth and development. BRs control important agronomic traits and responses to abiotic stresses. Through the signaling pathway, BRs control the expression of thousands of genes, resulting in a variety of biological responses. The key effectors of the BR pathway are two transcription factors (TFs): BRASSINAZOLE RESISTANT 1 (BZR1) and BRI1-EMSSUPPRESSOR 1 (BES1). Both TFs are phosphorylated and inactivated by the Glycogen synthase kinase 3 BRASSINOSTEROID INSENSITIVE2 (BIN2), which acts as a negative regulator of the BR pathway. In our study, we describe the functional characteristics of *HvGSK1.1*, which is one of the GSK3/SHAGGY-like orthologs in barley. We generated mutant lines of *HvGSK1.1* using CRISPR/Cas9 genome editing technology. Next Generation Sequencing (NGS) of the edited region of the *HvGSK1.1* showed a wide variety of mutations. Most of the changes (frameshift, premature stop codon, and translation termination) resulted in the knock-out of the target gene. The molecular and phenotypic characteristics of the mutant lines showed that the knock-out mutation of *HvGSK1.1* improved plant growth performance under salt stress conditions and increased the thousand kernel weight of the plants grown under normal conditions. The inactivation of HvGSK1.1 enhanced BR-dependent signaling, as indicated by the results of the leaf inclination assay in the edited lines. The plant traits under investigation are consistent with those known to be regulated by BRs. These results, together with studies of other *GSK3* gene members in other plant species, suggest that targeted editing of these genes may be useful in creating plants with improved agricultural traits.

## 1. Introduction

Brassinosteroids (BRs) are a class of plant steroid hormones that are essential for plant growth and development. BRs control important agronomic traits, such as grain yield [1,2,3,4,5] and responses to biotic and abiotic stresses [6,7]. Studies in various plant species treated with exogenous BR [8,9,10,11] and mutants with enhanced BR signaling [12,13] confirm the positive role of BRs in the regulation of processes that determine salt stress tolerance. Through the signaling pathway, BRs control the expression of thousands of genes, resulting in a variety of biological responses [14,15,16]. The key effectors of the BR pathway are two transcription factors (TFs): BRASSINAZOLE RESISTANT 1 (BZR1) and BRI1-EMSSUPPRESSOR 1 (BES1). Both TFs are phosphorylated and inactivated by the GSK3-like kinase BRASSINOSTEROID INSENSITIVE2 (BIN2) [17]. The phosphorylation of BZR1 and BES1 inhibits their nuclear localization and DNA-binding activity, thereby limiting their ability to regulate BR-dependent genes [18,19,20,21]. Glycogen synthase kinase 3 (GSK3), also known as the Shaggy-like kinase (named after the morphological phenotype of a *Drosophila melanogaster* GSK3-deficient mutant), is a highly conserved serine–threonine kinase present in all eukaryotes. In plants, GSKs are encoded by a multigene family [22,23] and have a significant impact on proteins that are involved in different pathways associated with development, hormone signaling, or stress responses [24,25]. *Arabidopsis* GSKs (AtSKs or ASKs) are encoded by a family of ten genes and represent the best-characterized plant GSKs [22,26,27]. AtSK21 (BIN2) is the first and most studied plant GSK3. It was identified as a negative regulator of the BR signaling pathway [20,21,28]. Out of the remaining AtSKs, at least seven (AtSK11, AtSK12, AtSK13, AtSK21, AtSK22, AtSK23, and AtSK31) function in BR-dependent regulation, as it was shown in genetic screens and the application of bikinin, the GSK3-specific inhibitor [17,29,30,31,32]. Through interactions with upstream regulators and by affecting the activity of various substrate proteins, GSKs are involved in the regulation of various cellular and developmental processes [23] and in the regulation of plant responses to biotic and abiotic stresses [24,33,34,35].

The involvement of plant GSKs in the response to salt stress has been widely reported, although the data are inconclusive and show that different members of the GSK3 proteins respond in opposite ways to salt treatment in different species. In *Arabidopsis*, the AtGSK1 and ASKα play a positive role in the regulation of salt stress tolerance. The overexpression of *AGSK1* increased plant resistance to sodium chloride (NaCl) stress and induced the expression of the NaCl stress-responsive genes [36], where ASKα regulated stress tolerance through the activation of Glc-6-phosphate dehydrogenase (G6PD), which was essential for maintaining the redox balance in cells [37]. Soybean (*Glycine max*) GSK3-like kinase GmSK2-8 was strongly induced under salt stress [38], and the heterologous overexpression of *GmBIN2* improved salt stress tolerance in *Arabidopsis* plants [39]. The overexpression of the alfalfa *MsK4* (*Medicago sativa Kinase 4*) and two GSK3s members in wheat (*Triticum aestivum*), *TaGSK1*, and *TaSK5* enhanced salt tolerance in *Arabidopsis* [40,41,42]. Moreover, in wheat, the *TaGSK1* gene was induced by NaCl stress, and its expression was stronger in salt-resistant lines than in salt-sensitive ones [43], indicating positive regulation of this trait.

On the contrary, certain members of plant GSK3s kinases were found to negatively regulate salt stress tolerance. In rice, knock-out mutants of *OsGSK1* showed increased tolerance to salt stress [44]. Our earlier research has shown that the RNAi-mediated silencing of barley (*Hordeum vulgare*) *HvGSK1.1* gene led to enhanced BR-dependent signaling, increased salt stress tolerance, and was correlated with higher seedling biomass and thousand kernel weight [45]. Gene silencing is a valuable tool for the study of gene function. However, it requires the continuous presence of the transgene, and, as our previous studies have shown, the level of silencing is not stable between different lines and generations. Opposite to this, CRISPR/Cas9-based editing leads to stable changes in the nucleotide sequence of the target gene change. The mutations are inherited and can be segregated from the T-DNA locus.

Given this, we used CRISPR/Cas9 genome editing technology to generate mutants of the *HvGSK1.1* gene, one of the GSK3/SHAGGY-like orthologs of barley [45,46]. This study aimed to generate genetically stable mutants of the *HvGSK1.1* gene and verify the phenotypic features observed in our previous studies after *HvGSK1.1* RNAi silencing [45]. We describe the molecular characteristics, phenotypic changes, and response to salinity stress of these plants. The most pronounced phenotypic characteristics of *HvGSK1.1* knock-out mutants were bigger biomass of seedlings grown under normal and salt stress conditions and greater thousand kernels weight compared to the WT control. The traits of the knock-out mutant lines of *HvGSK1.1* are consistent with those known to be regulated by BRs.

## 2. Results

### 2.1. Generation of HvGSK1.1 Barley Mutants Using CRISPR/Cas9-Mediated Genome Editing

**Figure 1:**
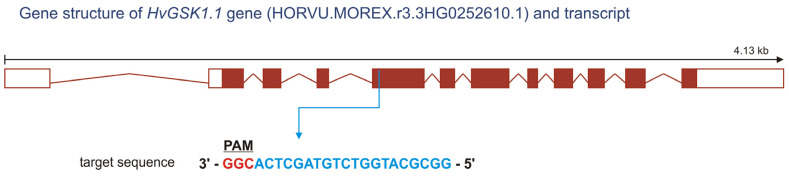
Schematic structure of the *HvGSK1.1* gene (adapted from Ensembl Plants) with the location of the target site. The nucleotide sequence of gRNA within the fifth exon is highlighted in blue. The protospacer adjacent motif (PAM) containing the CGG sequence is highlighted in red.

The gRNA sequence was complementary to the fifth exon of the target *HvGSK1.1* (Figure 1, Table S1) in the region encoding the Glycogen synthase kinase 3 catalytic domain.

**Figure 2:**
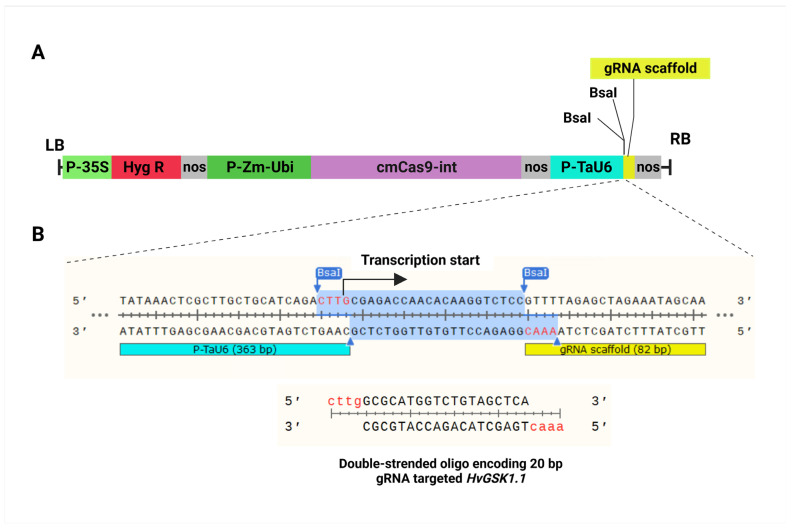
Schematic description of the CRISPR/Cas9-sgRNA construct of the pBract211cmCas9-sgRNA-gsk1.1 vector used for barley genome editing (**A**). The vector is a derivative of the pBract211-Cas [47]. LB, left border; P-35S, CaMV35S promoter; Hyg R, hygromycin resistance gene; nos, nopaline synthase terminator; P-Zm-Ubi, maize ubiquitin promoter; cmCas9-int, synthetic Cas9 nuclease gene with intron and nuclear localization signal, optimized for expression in monocots; P-TaU6, wheat U6 promoter; gRNA scaffold; *Bsa*I, *Bsa*I restriction sites between the U6 promoter and gRNA in opposite orientation; RB, right border. Scheme and nucleotide sequence of the gRNA cloning site in the pBract211cmCas9-sgRNA-gsk1.1 vector (**B**). After cutting with *Bsa*I, the shaded sequence is replaced by the designed oligo duplex. Complementary overhangs are highlighted in red.

The annealed oligos complementary to the gRNA, and 5′ overhang 5′-CTTG-3′ in the forward and 5′-AAAC-3′ in the reverse oligo were cloned into the delivery pBract211cmCas9-sgRNA vector between the U6 promoter and gRNA scaffold at the *Bsa*I cleavage site (Figure 2A,B). The RNA transcripts driven by the U6 promoter start with G; therefore, the target sequence had the pattern G(N)19NGG (Figure 2B).

**Figure 3:**
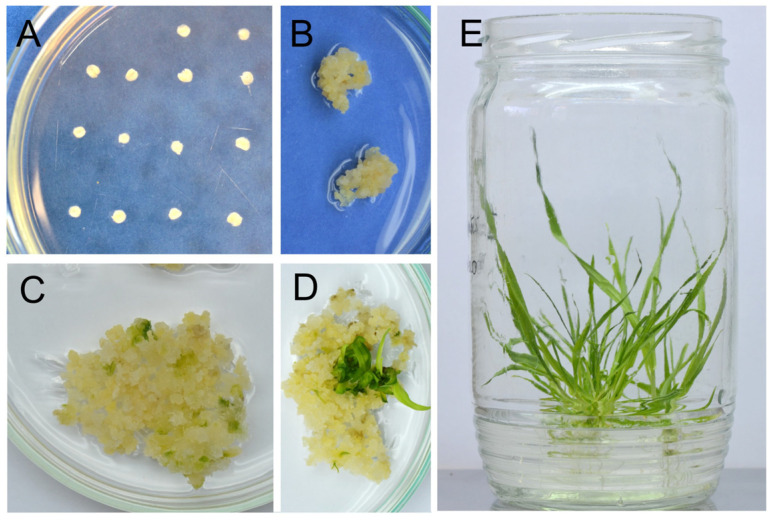
Representative pictures of the genetic transformation of immature barley embryos (cv. Golden Promise) and in vitro culture. (**A**) Barley immature embryos on a CI induction medium 1 day after isolation; (**B**) callus on a CI_sel medium containing hygromycin, one week after inoculation; (**C**) callus with regeneration centers on a TR_sel selection medium; (**D**) emerging plants on a TR_sel medium; (**E**) strong, rooted plants on a ½ MS medium after rooting on a Reg_sel selection medium.

A total of 1800 barley immature embryos (Figure 3A) were used for *Agrobacterium*-mediated transformation with the pBract211cmCas9-sgRNA-gsk1.1 vector. In vitro culture in a hygromycin-containing medium resulted in the regeneration of 57 T_0_ plants. Integration of T-DNA was confirmed in 50 plants, and the subsequent T7EI screening confirmed mutations of the target region in 29 plants. This represented an editing efficiency of 58% (Table 1, Figure 3).

The 19 randomly selected plants out of 29 T_0_ plants with the putative mutation were subjected to NGS, Illumina. In the analyzed group of the nineteen T_0_ mutant plants, six plants (31.6%) were heterozygous, three plants (15.8%) were biallelic, and ten plants (52.6%) were chimeric mutants (Table 2). The NGS analysis showed a wide variety of mutations (Table 2). As expected, most of the changes are represented by short InDels, which caused frameshift, premature stop codon, and premature translation termination. Two lines with six or nine bp deletion represented mutants with expected changes in the amino acid composition of HvGSK1.1 (Figure S2).

All edited T_0_ plants successfully set seeds. The three homozygous lines, #150, #17, and #29 from T_2_–T_3_ generation, derived from chimeric T_0_ plant #VIII.8 (Table 2), were selected for further analysis.

**Figure 4:**
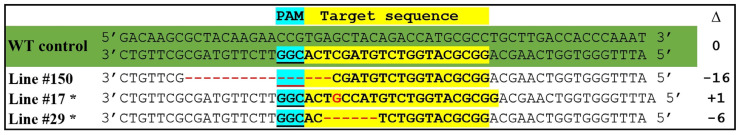
Alignment of *HvGSK1.1* target sequences from selected T_2_ homozygous lines after NGS (Illumina) sequencing. The sgRNA target sequences and the PAM motifs are highlighted in yellow and blue, respectively. Red letters indicate inserted nucleotides. Red dashed lines represent deleted nucleotides. Δ refers to changes in the CRISPR/Cas9 targeted sequence: 0, no change; − deletion; + insertion. * Segregants without T-DNA (Cas9-sgRNA) insertion.

Line #150 had sixteen bp deletions, line #17 had one bp insertion, and line #29 had six bp deletions (Figure 4). Lines #150 and #17 represented knock-out mutants, whereas #29 represented mutants with expected amino acid changes. Additionally, lines #17 and #29 represented segregants without T-DNA insertion and the Cas9-sgRNA cassette.

### 2.2. Leaf Inclination Biotest

The leaf inclination biotest is a semiquantitative assay used for the assessment of the biological activity of BRs [50] and BR-dependent signaling pathways [51], and it was adapted to barley [45]. We hypothesized that the knock-out mutation of *HvGSK1.1* would enhance the BR-dependent signaling. The level of the BR-dependent signaling could be estimated by observing the leaf inclination angles. Stronger signaling means bigger the inclination angles. Plant samples that were subjected to this biotest were treated with the 24-epibrassinolide (EBL) and bikinin, a compound that specifically blocked the activity of GSK enzymes, thereby promoting transduction in the BR pathway [17]. Leaf inclination angles were measured in three homozygous T_3_ mutant lines: line #150 and line #17, both represened knock-out mutants, and line #29, represented mutants with expected changes in the amino acid composition of HvGSK1.1.

The results of the leaf inclination angles were shown as relative values assuming a 1.0 value for the WT control and edited lines incubated in water.

**Figure 5:**
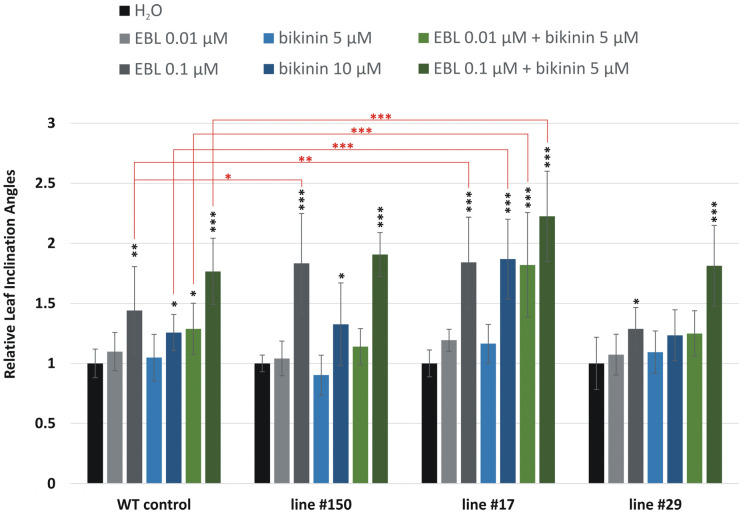
Relative values leaf inclination angles of WT plants and homozygous mutant lines of *HvGSK1.1* (T_3_ generation) after treatment with 24-epibrassinolide (EBL) and bikinin. Data represent the mean and standard deviation of at least six plants. Leaf inclination angles of the WT control and edited lines incubated in water (H_2_O) solutions were assumed to be 1.0. Black asterisks (*) indicate statistically significant changes within the same group between the variant sample and the same sample treated with H_2_O. Red asterisks (*) indicate statistically significant changes between the variant sample of the mutant line and the corresponding sample in the WT plant (as marked in the figure). Statistical significance changes are indicated: *^/^* *p* ≤ 0.05, **^/^** *p* ≤ 0.01, ***^/^*** *p* ≤ 0.001.

**Figure 6:**
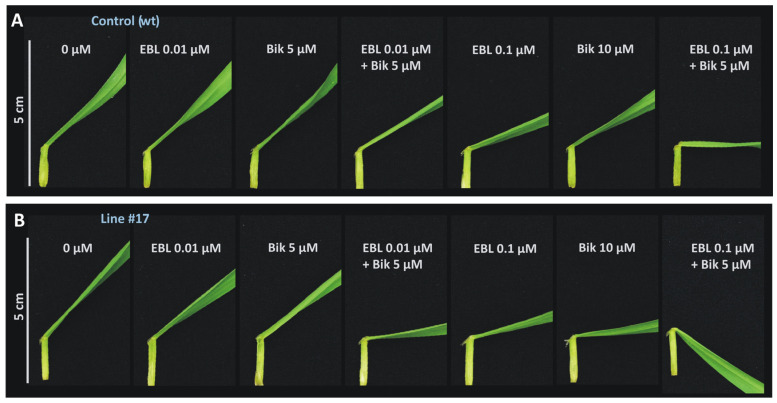
Representative images of leaf fragments treated with 24-epibrassinolide (EBL) and bikinin (Bik). (**A**) WT control; (**B**) line #17 (knock-out mutant of *HvGSK1.1*). The bar represents 5 cm.

The relative inclination angles of the WT control samples treated with EBL were 1.10 (SD ± 0.16) (for an EBL concentration of 0.01 μM) and 1.44 (SD ± 0.36) (EBL 0.1 μM). The relative inclination angles of WT samples treated with bikinin were 1.05 (SD ± 0.19) (bikinin 5 μM) and 1.26 (SD ± 0.15) (bikinin 10 μM). The relative inclination angles of the WT control samples after simultaneous treatment with both compounds, EBL and bikinin, were 1.29 (SD ± 0.21) (EBL 0.01 μM and bikinin 5 μM) and 1.77 (SD ± 0.28) (EBL 0.1 μM and bikinin 5 μM) (Figure 5 and Figure 6A).

The relative inclination angles of line #150 treated with EBL were 1.04 (SD ± 0.14) (for an EBL concentration of 0.01 μM) and 1.83 (SD ± 0.42) (EBL 0.1 μM). The relative values of samples treated with bikinin were 0.90 (SD ± 0.17) (bikinin 5 μM) and 1.33 (SD ± 0.34) (bikinin 10 μM). The relative values of this line after simultaneous treatment with both compounds, EBL and bikinin, were 1.14 (SD ± 0.15) (EBL 0.01 μM and bikinin 5 μM) and 1.91 (SD ± 0.18) (EBL 0.1 μM and bikinin 5 μM) (Figure 5).

The relative inclination angles of line #17 treated with EBL were 1.19 (SD ± 0.09) (for an EBL concentration of 0.01 μM) and 1.84 (SD ± 0.38) (EBL 0.1 μM). The leaf inclination angles of this line treated with bikinin were 1.16 (SD ± 0.16) (bikinin 5 μM) and 1.87 (SD ± 0.33) (bikinin 10 μM). The relative inclination angles of line #17 after simultaneous treatment with both compounds, EBL and bikinin, were 1.82 (SD ± 0.44) (EBL 0.01 μM and bikinin 5 μM) and 2.22 (SD ± 0.38) (EBL 0.1 μM and bikinin 5 μM) (Figure 5 and Figure 6B).

The relative inclination angles of line #29 treated with EBL were 1.07 (SD ± 0.17) (for an EBL concentration of 0.01 μM) and 1.29 (SD ± 0.18) (EBL 0.1 μM). The relative values of this line treated with bikinin were 1.09 (SD ± 0.18) (bikinin 5 μM) and 1.23 (SD ± 0.21) (bikinin 10 μM). The relative values of line #29 after simultaneous application of both compounds, EBL and bikinin, were 1.25 (SD ± 0.19) (EBL 0.01 μM and bikinin 5 μM) and 1.81 (SD ± 0.34) (EBL 0.1 μM and bikinin 5 μM) (Figure 5).

### 2.3. The hvgsk1.1 Knock-Out Has a Positive Effect on the Biomass of Plants Grown under Normal and Salinity Conditions

**Figure 7:**
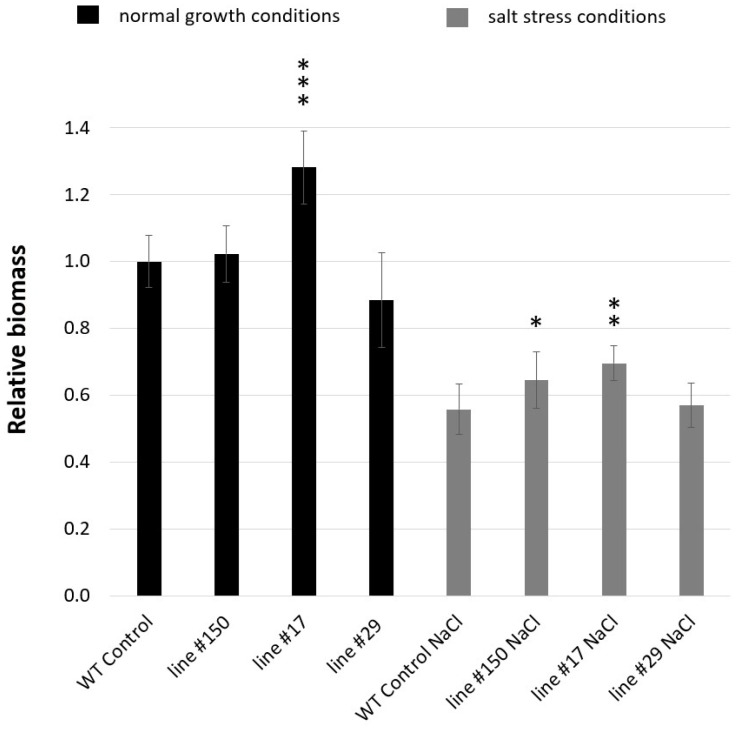
Relative biomass of the WT control and *HvGSK1.1* homozygous mutant lines of T_3_ generation grown under normal (Hoagland medium) and salt stress conditions (Hoagland medium supplemented with NaCl 150 mM). Data represent the mean and standard deviation of at least six plants grown under normal and salt-stressed conditions. The biomass of the WT control grown under normal conditions (Hoagland medium) was taken as 1.0. Significant differences between the WT control/WT control NaCl and the line grown under the same conditions are shown: * *p* ≤ 0.05, ** *p* ≤ 0.01, *** *p* ≤ 0.001.

**Figure 8:**
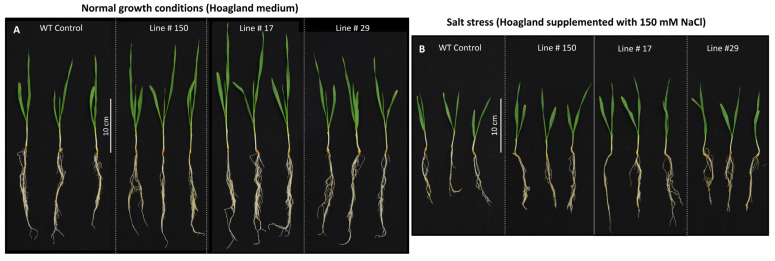
Representative pictures of 14-day-old seedlings of the WT control and the three mutant lines of *HvGSK1.1* grown under normal (Hoagland medium) (**A**) and salt stress conditions (Hoagland’s medium supplemented with 150 mM NaCl) (**B**). The bar represents 10 cm. The figure shows three seedlings from each of the tested lines out of a minimum of six plants analyzed, whose biomass is shown in Figure 7.

The relative biomass of *HvGSK1.1* knock-out mutant lines was greater than the WT control grown under the same conditions, and it was observed for both normal (Hoagland medium) and salt stress conditions (Hoagland medium supplemented with NaCl 150 mM) (Figure 7 and Figure 8). Under normal growth conditions, the relative biomass of line #150 was 1.02 (SD ± 0.11) and exceeded the biomass of the WT control by 2%. The relative biomass of line #17 was 1.28 (SD ± 0.08), exceeded the biomass of the WT control by 28% and had a high statistical significance of *p* ≤ 0.001. The relative biomass of line #29 was 0.88 (SD ± 0.14) and was 12% lower than the WT control (Figure 7 and Figure 8A).

The biomass of the plants grown under salt stress was lower compared with normal conditions. However, the relative values of the biomass of all three mutant lines grown under salt stress were bigger than the corresponding values of the WT control in the same conditions. The relative biomass WT control under salt stress was 0.56 (SD ± 0.08) of the biomass WT control under normal conditions. Respectively, the relative biomass of line #150 was 0.65 (SD ± 0.05), 0.70 in line #17(SD ± 0.08), and 0.57 in line #29 (SD ± 0.07) of the biomass WT control under normal conditions. Two knock-out mutant lines, #150 and #17, exceeded the WT control in the salt stress conditions by 16% and 25% respectively, with statistical significances of *p* ≤ 0.05 and *p* ≤ 0.01 (Figure 7 and Figure 8B).

### 2.4. Thousand Kernel Weight (TKW)

**Figure 9:**
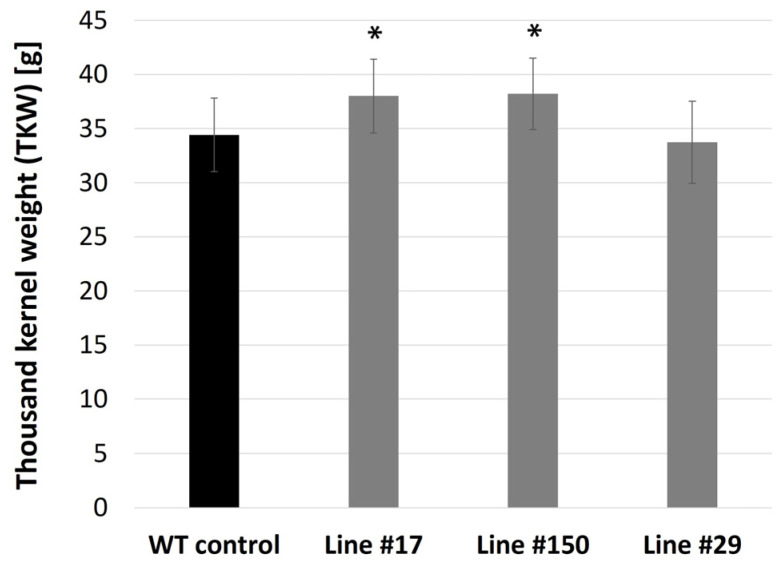
Thousand kernels weight (TKW) in the WT control and the three mutant lines of *HvGSK1.1* grown in soil in normal conditions. Data represent mean values and the standard deviation of at least eight biological replications. Significant differences between the control and the line grown under the same conditions are shown: * *p* ≤ 0.05.

**Figure 10:**
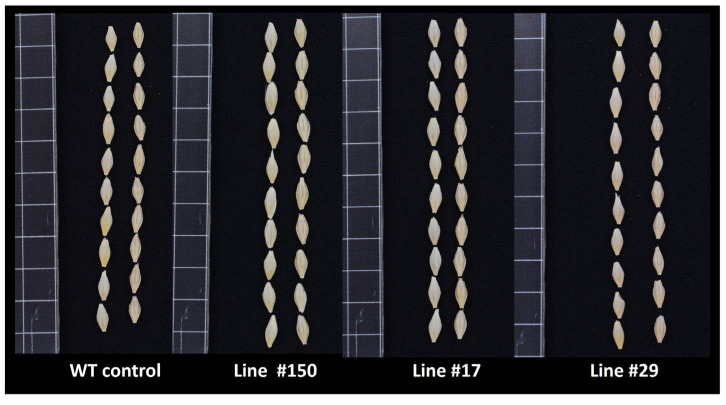
Representative pictures of the kernels of the non-transgenic WT control and the three mutant lines of *HvGSK1.1*. One grid (square) represents 1 cm.

The thousand kernel weight (TKW) of the WT control plants grown in soil under normal conditions was 34.42 g (SD ± 3.38) (Figure 9 and Figure 10). For the *hvgsk1.1* mutants (lines #150 and #17), the values were 38.21 g (SD ± 3.29) and 38.01 g (SD ± 3.41), respectively. The values were significantly higher (*p* ≤ 0.05) than in the control by 11% and 10.42%, respectively. In contrast, the TKW of line #29 was 33.73 g (SD ± 3.79), which was 2.02% lower than the WT control (Figure 9 and Figure 10).

## 3. Discussion

The aim of the study was to obtain and characterize mutant lines of the *HvGSK1.1* gene to confirm the role of this gene in BR-dependent signaling and involvement in the regulation of salt stress tolerance and yield-related traits in barley plants.

The *HvGSK1.1* gene is one of the barley GSK3/SHAGGY-like orthologs [46] and was the first of the barley GSK-encoding genes identified by our team [45]. As mentioned in the introduction, the *HvGSK1.1* gene was the subject of our previous study in which we characterized the effect of the *HvGSK1.1* silencing in barley plants using RNAi technology. The silencing of *HvGSK1.1* enhanced BR-dependent signaling in the plants. The *HvGSK1.1*-silenced lines were characterized by better growth under salt stress conditions, a bigger seedling biomass, and a bigger thousand kernel weight. However, the obtained results also showed that the silencing of the target gene was maintained at different levels in individual lines and was not stable in the next generation [45]. This led us to generate mutants of the *HvGSK1.1* gene using the CRISPR/Cas9 system.

CRISPR/Cas9 genome editing has become a powerful tool for functional plant genomics, which allows for the precise mutagenesis of a specific region of the genome, and the resulting mutations are stably inherited. The use of RNA-guided Cas9 endonucleases, adapted from the immune mechanisms of prokaryotes based on CRISPR/Cas9, is a highly efficient genome editing system that was successfully applied in barley plants [47,52,53,54]. To obtain mutants of the *HvGSK1.1* gene, we used the RNA-guided Cas9 endonuclease expressed from the Cas9-sgRNA cassette, which was introduced into barley plants via stable *Agrobacterium*-mediated transformation using the binary vector pBract211_cmCas9-sgRNA. The genetic transformation of immature barley embryos resulted in 50 T_0_ plants, and 29 of them showed a mutation in the target gene, indicating a high 58% efficiency of the mutation in the edited region. The first attempts of RNA-guided Cas9-based editing in monocot species showed lower frequencies, ranging from 8% to 23% [54,55,56]. Currently, following system optimization, the efficiencies range from 60% to 100% [47,57,58,59,60].

Barley is the fourth most important cereal crop in the world. Compared to other cereals, such as wheat, rice, and maize, barley is characterized by a higher tolerance to salt and drought, which enables it to adapt to the environment and be widely distributed worldwide. In addition, barley grains are rich in β-glucan and tocols, which benefit human health [61,62]. Barley is a diploid plant, and after integration of the CRISPR/Cas9 system, five types of genotypes can be expected in T_0_ transgenic plants: homozygotic, biallelic and heterozygotic mutants, chimeric plants, and non-mutated WT plants. The NGS sequencing (Illumina) of 19 T_0_ plants with a confirmed mutation (based on T7EI digestion) showed that 31.6% of the mutants were heterozygous and 15.8% were biallelic. In this group, 52.6% of the plants were chimeric (Table 2). Although the numbers provided in other reports are different, the pattern and types of mutants in T_0_ are compatible with Zhang et al. [63].

BRs are involved in the regulation of leaf inclination angle in rice [64]. The degree of leaf blade inclination is a good indicator for the physiological testing of BR concentration in vivo [65]. BR-deficient and -insensitive mutants show erect leaves (low degree inclination), whereas the overexpression of BR biosynthetic or signaling genes elevates leaf inclination [66,67,68]. The mechanism in this process depends on the direct regulation of cell division of abaxial sclerenchyma cells in rice lamina joints [69] and cell elongation on the adaxial side of the blade joint [70] through BES1/BZR1-related genes. The number of abaxial sclerenchyma cells in rice lamina joints (LJs) is closely related to leaf erectability, and BR signaling tightly regulates their proliferation [69]. BRs, which induce cell elongation on the adaxial side of the blade joint, promote changes in leaf inclination angle [70].

We expected that the knock-out mutation of *HvGSK1.1* would enhance BR-dependent signaling. The level of this signaling could be estimated by checking leaf inclination angles. The observation is that stronger signaling leads to bigger inclination angles, which is the basis for leaf inclination bioassay. 24-epibrassinolide (EBL) treatments mimic the reaction for BR, while bikinin, which is an inhibitor of the GSK [29], mimics the enhancement of BR signaling, which is an expected result of the *HvGSK1.1* mutation. Our early results showed that higher concentrations of EBL (between 0.1 μM and 2 μM) and bikinin (10 μM) were associated with larger leaf inclination angles. The lowest concentrations that induced bending of the leaf blade were 0.01 μM of EBL and 5 μM of bikinin [45]. In the present manuscript, to observe differences in the sensitivity of the *HvGSK1.1* mutant lines and the WT control to the exogenous EBL and bikinin treatment, we used the following concentrations of these compounds: 0.01 μM of EBL—the lowest concentration of EBL, which induced bending of the leaf blade; 0.1 μM of EBL—a higher concentration, which activated significant leaf blade bending; 5 µM of bikinin—the lowest concentration of bikinin, which induced bending of the leaf blade; and 10 µM of bikinin—the highest concentration, which caused a strong deflection of the leaf blade. In other variants, the two compounds were combined and used together: EBL 0.01 μM and bikinin 5 µM—the lowest concentrations of the two compounds; and EBL 0.1 μM and bikinin 5 µM—a higher concentration of EBL and the lowest concentration of bikinin. Our results have shown that 0.1 μM of 24-epibrassinolide (EBL) and 10 μM bikinin are associated with larger leaf inclination angles. In seedling fragments treated separately with EBL or bikinin (Figure 5 and Figure 6), this effect was clearly visible. Co-treatment with 0.01 μM EBL and 5 μM bikinin showed a stronger effect than the effect of each compound applied alone. The changes observed after treatment with EBL and bikinin of the WT control and *hvgsk1.1* mutants indicated that the knock-out mutation resulted in the enhanced response to exogenously applied EBL in a manner-like treatment with exogenously applied bikinin. *hvgsk1.1* line #17 was more sensitive to treatment with EBL or bikinin than the WT control. The treatment of line #17 with 0.1 µM EBL and 10 µM bikinin increased leaf angles by 84% and 87%, respectively, compared to the treatment of this line with water (H_2_O), while the treatment of the WT control with 0.1 µM EBL and 10 µM bikinin increased leaf angles by 44% and 26%, respectively, compared to the treatment of the WT control with water (H_2_O) (Figure 5 and Figure 6). Co-treatment with both compounds, EBL and bikinin, of *hvgsk1.1* line #17 increased leaf inclination angles by 82% after simultaneous treatment with EBL (0.01 µM) and bikinin (5 µM) and 122% after simultaneous treatment with EBL (0.1 µM) and bikinin (5 µM) compared to the treatment of this line with water (H_2_O), whereas the WT control co-treatment with both compounds, EBL and bikinin, increased leaf inclination angles by 29% after simultaneous treatment with EBL (0.01 µM) and bikinin (5 µM) and 77% after simultaneous treatment with EBL (0.1 µM) and bikinin (5 µM) compared to the treatment of the WT control with water (H_2_O) (Figure 5 and Figure 6). *hvgsk1.1* line #150 was more sensitive to treatment with 0.1 μM EBL than the WT control. The treatment of line #150 with 0.1 μM EBL increased leaf angles by 83% compared to the treatment of this line with water (H_2_O), whereas the treatment of the WT control with 0.1 μM EBL increased leaf angles by 44% compared to the treatment of the WT control with water (H_2_O) (Figure 5). The statistically significant increase of leaf inclination angle of the knock-out mutants (line #17 and line #150) compared to the WT control after EBL and bikinin treatment allowed us to conclude that the knock-out mutation of the *HvGSK1.1* enhanced BR-dependent signaling in these mutants. 

A notable feature of the knock-out mutant lines (lines #17 and #150) grown under normal and salt stress conditions was a bigger seedling biomass compared to the WT control (Figure 7 and Figure 8). Under normal growth conditions, the relative biomass of lines #150 and #17 exceeded the biomass of the WT control by 2% and 28%, respectively. Under salt stress conditions, the relative biomass of lines #150 and #17 was greater than the WT control under the same conditions and exceeded by 16% and 25%, respectively. Similar results have been reported in rice, where greater plant weights have been found after EBL treatment under normal and salt-stressed conditions [8]. Several other studies reported similar results of growth stimulation and salt stress alleviation by the exogenous application of EBL [10,71,72]. According to Perez-Perez et al. [73], the observed phenotypic effects of exogenous BR application may be related to auxin-dependent pathways. The increased biomass found in *HvGSK1.1* knock-out mutant lines is in agreement with findings reported in moss bamboo, where *PeGSK1* acts as a negative regulator of cell growth. The overexpression of *PeGSK1* in *Arabidopsis* caused significant growth arrest phenotypes, including dwarfism, small leaves, reduced cell length, and impaired petiole elongation [74].

The importance of GSK3s in the regulation of salt stress responses in plants was reported in many articles. However, these studies indicated that different members of GSK3-encoding genes had different, sometimes opposite roles in salt stress response. In *Arabidopsis*, *AtGSK1* plays a positive role in the regulation of salt stress tolerance [36]. Similarly, *GmSK2-8* and *GmBIN2* in soybean [38,39], *OsSK41*/*OsGSK5* in rice [75], *MsK4* in *Medicago sativa* [41], and at least two types of wheat, *TaGSK1* [40] and *TaSK5* [42], positively affected salinity tolerance. In contrast, certain members of plant GSK3 kinases were found to negatively regulate salt stress tolerance. In rice, the knock-out mutants of *OsGSK1* showed elevated tolerance to salt stress [44]. The heterologous overexpression of *StSK21* (*Solanum tuberosum*) [76] and *AgSK*s (*Apium graveolens*) [77] led to increased salt stress sensitivity in *Arabidopsis*. From the above results, we can see that some of the GSK3 kinases function as positive while others are negative regulators of plant salt tolerance. 

Other important traits found in the knock-out mutant lines of the *HvGSK1.1* were larger kernels and a larger thousand kernel weight (TKW) compared to the WT control (Figure 9 and Figure 10). The values of TKW in the knock-out mutant lines #150 and #17 were significantly higher than the WT control by 11% and 10.42%, respectively. These findings are consistent with data from the literature showing that productivity-related traits are stimulated by BR and BR-dependent signaling [78,79] and are in line with our previous findings, in which the silencing of the *HvGSK1.1* gene correlates with a higher thousand kernel weight [45]. These results also agree with other researchers who have shown that a mutation in the grain size-associated locus (GL2) in rice activates BR-dependent responses and leads to an increase in rice grain weight [80]. Our results also agree with findings reported by Liu et al. [3], in which rice GSK3/SHAGGY-LIKE KINASE1 (GSK1)-GSK4 mutants have enlarged grains. In contrast, the TKW of line #29 was 2.02% lower than the WT control (Figure 9 and Figure 10). Moreover, the relative biomass of line 29 was 12% less than the WT control under normal growth conditions (Figure 7 and Figure 8A) and was not significantly different under salt stress conditions (Figure 7 and Figure 8B). Line #29 had a six bp deletion (Figure 4) and represented a mutant with an expected two amino acid deletion of HvGSK1.1 (Figure S2). This is unconfirmed, but we speculate that the altered amino acid sequence could result in the synthesis of at least partially active HvGSK1.1 protein. We cannot exclude that a protein with two amino acid deletions could retain some of its biological activity. This line will be the subject of further research. The other two lines (#17 and #150) represent the knock-out mutants.

The phenotypic characteristics of the *HvGSK1.1* gene knock-out mutant lines described in the current publication are compatible with those described in our previous work analyzing plants after *HvGSK1.1* silencing [45]. The homozygous mutant lines obtained in this work are genetically stable. This means that mutations resulting from the editing are inherited in subsequent generations. Furthermore, we have also obtained two mutant lines (#17 and #29) that do not contain T-DNA insertion, making the resulting mutants indistinguishable from those mutants where the mutation occurred naturally. In conclusion, our results show that the barley knock-out mutant lines of the *HvGSK1.1* have improved growth performance under salt stress conditions. This suggests that *HvGSK1.1* is a negative regulator of salt tolerance in barley. The characteristics of barley lines with inactive HvGSK1.1 are consistent with the phenotype of plants with enhanced BR signaling. These results, together with studies of other members of the GSK3 gene family, suggest that targeted editing of these genes may be useful in generating plants with improved agricultural traits, particularly in the face of climate change.

## 4. Materials and Methods

### 4.1. Plant Material and Growth Conditions

The plant source for all experiments was the barley (*Hordeum vulgare* L.) cultivar Golden Promise. Plants were grown in soil in a controlled environment chamber with a 16 h photoperiod, 18 °C day and 12 °C night temperatures, and 350 μmol/m^2^/s light intensity provided by fluorescent lamps. The plants were watered twice a week and fertilized once a week with the multi-component soil fertilizer Florovit ( INCO GROUP, Warsaw, Poland) according to the manufacturer’s instructions. The same conditions were applied to regenerated T_0_ plants. All generations analyzed were grown under the same conditions as the donor plants. Phenotype characterization was performed using homogeneous seed material of the T_3_ generation.

### 4.2. Construction of the RNA-Guided Cas9 Vector and Agrobacterium-Mediated Transformation of Barley

The sequence of guide RNA (gRNA) specific to the *HvGSK1.1* and the possible off-targets were designed using the web-based CRISPOR online tool (http://crispor.tefor.net, accessed on 1 March 2022) and barley (*Hordeum vulgare* L.) reference genome (Hv IBSC PGSB v2) [81]. 

The cDNA complementary to the gRNA was synthesized (Genomed, Poland) as two complementary sequences, 5′-CTTG(N)19-3′ (forward oligo) and 5′-AAAC(N)19-3′ (reverse oligo), with 5′ and 3′ overhangs complementary to the *Bsa*I cleavage site needed for cloning in the destination vector (Table S1). The two annealed oligos were ligated between the U6 promoter and gRNA scaffold at the *Bsa*I cleavage site (Figure 2) of the destination vector pBract211cmCas9-sgRNA (Figure S1). The vector is a derivative of the pBract211-Cas [47] and is a gift from Professor A. Lyznik (unpublished data). The pBract211cmCas9-sgRNA vector contains (i) a synthetic Cas9 gene (based on the native sequence of *Streptococcus pyogenes* with codon usage optimized for expression in monocots) driven by the maize ubiquitin promoter; (ii) an sgRNA cassette consisting of the wheat U6 RNA promoter and the sgRNA sequence with a transcription termination signal; and (iii) a hygromycin resistance gene (*hptII*) as a selection marker for the regenerated plants. The Cas9-sgRNA cassette was amplified and verified by Sanger sequencing. The final construction of the binary pBract211cmCas9-sgRNA vector was electroporated into an *Agrobacterium tumefaciens* AGL1 strain containing a pSoup helper plasmid [82]. 

Immature barley (cv. Golden Promise) embryos, inoculated with the AGL1 strain carrying the pBract211cmCas9-sgRNA, were cultured in vitro according to the methods of Harwood et al. [83,84]. Regenerated plants were planted in soil and grown under the same conditions as the barley donor plants. Non-transgenic in vitro regenerated plants, referred to as the WT control, were used as controls for all phenotypic and molecular analyses in this study.

### 4.3. Genotype Analysis of Regenerated Plants

Genomic DNA (gDNA) was isolated from the leaves of T_0_–T_3_ plants using a modified CTAB method [85]. Putative transgenic plants were verified by the PCR amplification of T-DNA fragments using primers *hpt-F-205* and *hpt-R-205* (Table S1) specific for the *hptII* gene. 

To detect mutations in the target gene, transgenic plants were analyzed using T7 Endonuclease I (T7EI). For this purpose, the target region was amplified by PCR using primers *gsk1.1_Ontarg_L* and *gsk1.1_Ontarg_R* (Table S1) and Q5 hot-start polymerase (New England Biolabs, Ipswich, MA, USA). After amplification, 10 μL samples of the PCR mixtures of the tested and WT plants were mixed in a 1:1 ratio to form the heteroduplexes of amplicons, and the heteroduplex mixture was subjected to enzyme digestion with T7EI (New England Biolabs, Ipswich, MA, USA) according to protocol described by [86]. The digested amplicons were separated on 3% agarose gels (Micropor Delta, Prona, Madrid, Spain) and visualized on a Kodak Gel Logic 200 Imaging System. The same analysis was performed for predicted off-target sites.

### 4.4. NGS Sequencing

Amplicons of the edited *HvGSK1.1* region obtained by PCR reaction using specific primers *ADAPTgsk1.1F* and *ADAPTgsk1.1R* (Table S1) extended by Illumina platform-specific adapter sequences were subjected to Next Generation Sequencing (NGS). 

Library preparation and indexing (using the Nextera XT Index Kit, Illumina, San Diego, CA, USA) of the analyzed samples were performed according to the protocol developed for metagenomic libraries: https://emea.illumina.com/content/dam/illumina-support/documents/documentation/chemistry_documentation/16s/16s-metagenomic-library-prep-guide-15044223-b.pdf (Illumina), which can be adapted to any amplicon. Sequencing was performed on second-generation sequencer, a MiSeq sequencer (Illumina), using MiSeq Reagent Nano Kit v2 at 500 cycles (Illumina, San Diego, CA, USA). Files were obtained in fastq format and analyzed on the GALAXY platform, https://usegalaxy.org/ (accessed on 15 June 2023), using the bioinformatics tools required for analysis.

### 4.5. Leaf Inclination Test

The leaf inclination bioassay was adapted from the protocol of [47] and modified for barley seedlings according to [45]. Shoot fragments with the first leaf taken from nine-day-old seedlings were incubated for 72 h in the solutions containing 24-epibrassinolide (EBL) (OlChemIm, Olomouc, Czech Republic) and bikinin (OlChemIm, Olomouc, Czech Republic). Our early results showed that higher concentrations of EBL (between 0.1 μM and 2 μM) and bikinin (10 μM) were associated with larger leaf inclination angles, and the lowest concentrations that induced bending of the leaf blade were 0.01 μM of EBL and 5 μM of bikinin [45]. To determine the differences in the sensitivity of the mutant lines and the WT control compared to the exogenous treatment with EBL and bikinin, the following concentrations of these compounds were used:

0.01 μM of EBL—the lowest concentration of EBL to induce bending of the leaf blade;
0.1 μM of EBL—a higher EBL concentration with significant bending of the leaf blade;
5 µM of bikinin—the lowest concentration of bikinin to induce bending of the leaf blade; 10 µM of bikinin—the highest concentration of bikinin, tested in barley, which induced strong deflection of the leaf blade.
In other variants, both components were combined:
EBL 0.01 μM and bikinin 5 µM co-treated together—the lowest concentration of the two compounds;
EBL 0.1 μM, and bikinin 5 µM—a higher concentration of EBL and the lowest concentration of bikinin.
The former compound, which specifically blocks the activity of GSK enzymes, was used to promote the BR-dependent signaling pathway. After incubation, the leaf inclination angles were assessed using samples from at least six plants representing independent biological replications. The results were shown as relative changes in the angle of the analyzed sample versus the angle of the same sample incubated in water.

### 4.6. Biomass of Plants Grown in Normal and Salt Stress Conditions

Seeds from the edited lines and the WT control were surface sterilized with a 4% sodium hypochlorite, imbibed at 4 °C for 48 h, and germinated at 21 °C for 72 h. Seedlings were placed on filter paper, tightly rolled, and saturated with Hoagland solution [87] (normal conditions) or Hoagland supplemented with 150 mM NaCl (salt stress conditions) and placed in a growth chamber under the same conditions as the donor plants. The 14-day-old seedlings grown under normal and salt stress conditions were used for biomass quantification. At least six seedlings (independent biological replicates) representing the edited plants and the WT control were used to assess the biomass of plants grown under normal and salt stress conditions.

### 4.7. Thousand Kernel Weight

Thousand kernel weight (TKW) was calculated from kernels collected from at least eight plants of the edited lines and the WT control grown in soil under the same conditions as the donor plants.

### 4.8. Statistical Analyses

The statistical significance of differences was analyzed using an ANOVA test followed by an LSD post hoc test (STATISTICA v.10.0, StatSoft). Differences were considered statistically significant at *p* ≤ 0.05, *p* ≤ 0.01, or *p* ≤ 0.001.

## Figures and Tables

**Table 1:** Regeneration, transformation, and editing efficiencies of barley cv. ‘Golden Promise’ after genetic transformation with the pBract211cmCas9-sgRNA-gsk1.1 vector.

Regeneration Efficiency, Transformation Plant Selection, and Editing Efficiency (CRISPR/Cas9)		
Immature embryos	Inoculated	1800
Number of plants	Obtained after regeneration in a medium containing hygromycin	57
	With confirmed integration of T-DNA	50
	With the mutation in the target region (based on T7EI analysis)	29
Efficiency	Transformation	3.17%
	Selection	87.72%
	Editing	58%

**Table 2:** Types of mutations detected in selected T_0_ transgenic plants by NGS of target site amplicons. wt, wild type; het, heterozygote; chi, chimeric; bi-a, bi-allelic, − deletion; + insertion. Analyses were performed using the IGV [48] and Galaxy [49].

No.	Line	Mutation Types	Denotation
1	VIII.8	−6 bp, −16 bp, +1 bp, +1 bp, wt,	chi
2	2	−6 bp, wt,	het
3	4	−14 bp, +1 bp	bi-a
4	5	+1 bp, −16 bp, −14 bp, wt,	chi
5	7	+1 bp, −16 bp, wt,	chi
6	8	−6 bp, −1 bp, −16 bp, wt,	chi
7	9	−5 bp, +1 bp	bi-a
8	22	−16 bp, +1 bp, −6 bp, wt,	chi
9	26	−8 bp, +1 bp, wt,	chi
10	27	−28 bp, −10 bp, +1 bp	chi
11	28	−9 bp, +1 bp, wt,	chi
12	29	−2 bp, wt,	het
13	32	−5 bp, wt,	het
14	34	−8 bp, wt,	het
15	38	−13 bp, −16 bp, +1 bp, wt,	chi
16	39	−14 bp, wt,	het
17	41	−1 bp, +1 bp,	bi-a
18	47	−16 bp, wt,	het
19	49	−8 bp, −16 bp, wt,	chi

## Data Availability

All data are contained within the article and Supplementary Materials files.

## Supplementary Materials

The following supporting information can be downloaded at https://www.mdpi.com/article/10.3390/ijms25020998/s1.
