# Supplementary material for: HvGSK1.1 Controls Salt Tolerance and Yield through the Brassinosteroid Signaling Pathway in Barley
Source: Int J Mol Sci. 2024 Jan 13;25(2):998. doi: 10.3390/ijms25020998 (PMC10815662; doi:10.3390/ijms25020998)
Supplement: Supplementary file 1 [file ijms-25-00998-s001.zip › ijms-2758551-supplementary.pdf]

Supplementary Materials:

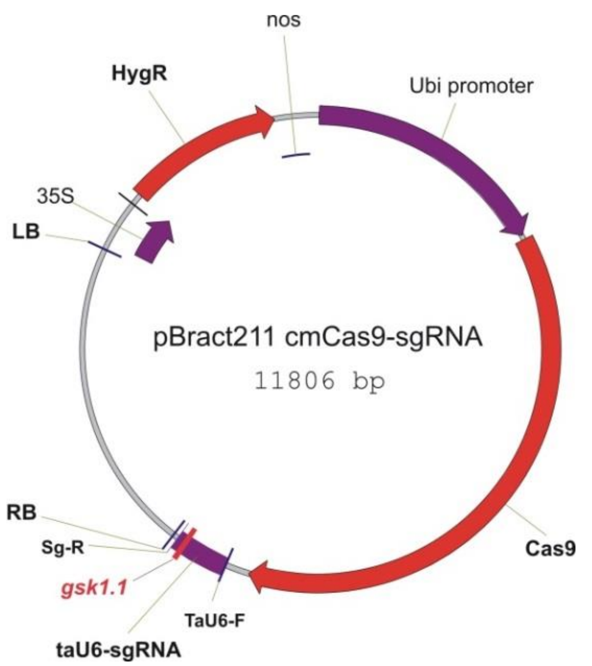

Figure S1. Scheme of the destination vector pBract211cmCas9-sgRNA. The vector pBract211cmCas9-sgRNA contains i) a synthetic Cas9 gene (based on the native sequence of *Streptococcus pyogenes* with codon usage optimized for expression in monocots) driven by the maize ubiquitin promoter; ii) an sgRNA cassette consisting of the wheat U6 RNA promoter, *gsk1.1* gRNA, and transcription termination signal, and amplification primers TaU6-F and Sg-R are indicated; iii) a hygromycin resistance gene (*hptII*) as a selection marker for the regenerated plants.

Table S1. List of the PCR primers and oligonucleotides that were used in this study.

Purpose	Symbol	Sequence	Oligos/Amplicon Length (bp)
oligos for cloning *HvGSK1.1* sgRNA HORVU.MOREX.r3.3HG0252610	oligo1_gsk1_1-Fw	5'-*cttg*GCGCATGGTCTGTAGCTCA-3'	23
	oligo1_gsk1_1-Re	5'-*aaac*TGAGCTACAGACCATGCGC-3'	23
PCR primers flanking the *hptII* gene	hpt-F-205	5′-GACGGCAATTTCGATGATG-3′	205
	hpt-R-205	5′-CCGGTCGGCATCTACTCTAT-3′	
PCR primers flanking the cloning site in the pBract211_cmCas9-sgRNA-gsk1.1 vector	TaU6 – F	5′-GACCAAGCCCGTTATTCTGA-3′	375
	Oligo gsk1.1- R	5′-AAACTGAGCTACAGACCATGCGC-3′	
PCR primers flanking the TaU6sgRNA cassette	TaU6 – F	5′-GACCAAGCCCGTTATTCTGA-3′	465 – 467
	Sg – R	5′- AAAAAAAGCACCGACTCGGTGCCAC-3′	
PCR primers flanking the *HvGSK1.1* target site sequence (HORVU.MOREX.r3.3HG0252610)	gsk1.1_Ontarg_L	5′-CCTGTGGCAAACTCCAATGC-3′	367
	gsk1.1_Ontarg_R	5′-CCTTCAGGACAAGCGCTACA-3′	
PCR primers flanking the *HvGSK1.1* target site sequence (HORVU.MOREX.r3.3HG0252610) extended by Illumina platform-specific adapters	ADAPTgsk1.1F	5′-TCGTCGGCAGCGTCAGATGTGTATAAGAGACAGGGGTGTA CCTGGAGCTTGTG-3′	359
	ADAPTgsk1.1R	5′-GTCTCGTGGGCTCGGAGATGTGTATAAGAGACAGGCATGCGCTGGTTCATCTTG-3′	
PCR primers flanking the Off-targets HORVU2Hr1G029120	Off_ 9120F	5′-TCGTCGGCAGCGTCGATCAATGTGCTCGGCGTTG-3′	160
	Off_ 9120R	5′-GTCTCGTGGGCTCGGAACAACTTCACCTCCGGCTT-3′	

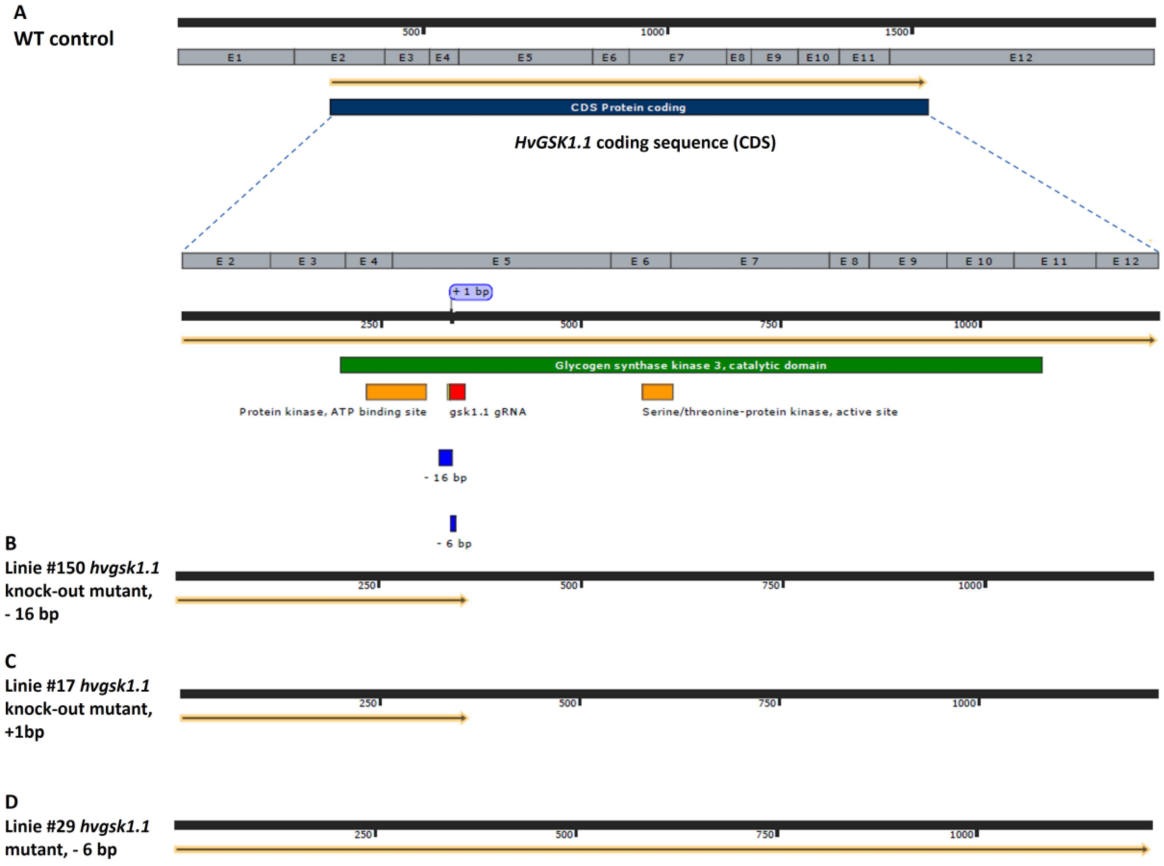

Figure S2. Diagram showing the structure of the *HvGSK1.1* transcript, CDS sequence, and GSK1.1 protein length WT (**A**), truncated (**B**, **C**), and with the expected deletion of two amino acids (**D**).

A Structure of *HvGSK1.1* transcript, E1-E12 exons, CDS (dark blue) and ORF (orange arrow), GSK3 catalytic domain (green color), protein kinase, ATP binding site, serine/threonine, protein kinase active site (orange color), gsk1.1 gRNA (red), PAM (yellow), mutations in the target site: deletion of 16 nt (-16 bp), insertion of 1 nt (+1bp), and deletion of 6 nt (-6 bp) (highlighted in blue).

B Predicted change in HvGSK1.1 protein length in the #150 knockout mutant line.

C Predicted change in HvGSK1.1 protein length in the #17 knockout mutant line.

D Predicted changes in HvGSK1.1 protein length in the mutant with two amino acid deletions in line #29.
